# Functional Analysis Helps to Define *KCNC3* Mutational Spectrum in Dutch Ataxia Cases

**DOI:** 10.1371/journal.pone.0116599

**Published:** 2015-03-10

**Authors:** Anna Duarri, Esther A. R. Nibbeling, Michiel R. Fokkens, Michel Meijer, Melissa Boerrigter, Corien C. Verschuuren-Bemelmans, Berry P. H. Kremer, Bart P. van de Warrenburg, Dennis Dooijes, Erik Boddeke, Richard J. Sinke, Dineke S. Verbeek

**Affiliations:** 1 Department of Genetics, University of Groningen, University Medical Center Groningen, Groningen, The Netherlands; 2 Department of Medical Physiology, University of Groningen, University Medical Center Groningen, Groningen, The Netherlands; 3 Department of Neurology, University of Groningen, University Medical Center Groningen, Groningen, The Netherlands; 4 Department of Neurology, Radboud University Medical Center, Nijmegen, The Netherlands; 5 Department of Medical Genetics, University Medical Center Utrecht, Utrecht, The Netherlands; University Hospital S. Maria della Misericordia, Udine, ITALY

## Abstract

Spinocerebellar ataxia type 13 (SCA13) is an autosomal dominantly inherited neurodegenerative disorder of the cerebellum caused by mutations in the voltage gated potassium channel *KCNC3*. To identify novel pathogenic SCA13 mutations in *KCNC3* and to gain insights into the disease prevalence in the Netherlands, we sequenced the entire coding region of *KCNC3* in 848 Dutch cerebellar ataxia patients with familial or sporadic origin. We evaluated the pathogenicity of the identified variants by co-segregation analysis and *in silico* prediction followed by biochemical and electrophysiological studies. We identified 19 variants in *KCNC3* including 2 non-coding, 11 missense and 6 synonymous variants. Two missense variants did not co-segregate with the disease and were excluded as potentially disease-causing mutations. We also identified the previously reported p.R420H and p.R423H mutations in our cohort. Of the remaining 7 missense variants, functional analysis revealed that 2 missense variants shifted Kv3.3 channel activation to more negative voltages. These variations were associated with early disease onset and mild intellectual disability. Additionally, one other missense variant shifted channel activation to more positive voltages and was associated with spastic ataxic gait. Whereas, the remaining missense variants did not change any of the channel characteristics. Of these three functional variants, only one variant was *in silico* predicted to be damaging and segregated with disease. The other two variants were *in silico* predicted to be benign and co-segregation analysis was not optimal or could only be partially confirmed. Therefore, we conclude that we have identified at least one novel pathogenic mutation in *KCNC3* that cause SCA13 and two additionally potential SCA13 mutations. This leads to an estimate of SCA13 prevalence in the Netherlands to be between 0.6% and 1.3%.

## Introduction

Spinocerebellar ataxia type 13 (SCA13) is an autosomal dominantly inherited neurodegenerative disorder characterized by atrophy of the cerebellum, especially the vermis, leading to a cerebellar syndrome with dysarthria and nystagmus. It is sometimes accompanied by pyramidal signs, epilepsy, auditory deficits, and mild intellectual disability [1–5]. Disease onset varies from early childhood, with delayed motor and cognitive skills acquisition, to late-onset, but the course is always very slowly progressive. The disease is caused by missense mutations in the *KCNC3* gene, which encodes the voltage-gated potassium channel subfamily C member 3, Kv3.3 [2,6].

The physiological role of Kv3.3 channels in the cerebellum is well known. Purkinje cells (PC) express Kv3.3 in both soma and dendrites [7–10], and plays a crucial role in the PC spikelets repolarization and shaping of the complex spike [11,12]. Kv3.3 forms tetrameric heterocomplexes with other Kv3 subunits to form a functional channel [13,14] and this has been implicated in A-type potassium currents that enable neurons to fire action potentials at high-frequencies [15].

So far, only three disease-causing mutations have been reported in *KCNC3*: p.R420H, p.R423H and p.F448L [2,6]. Functional analysis showed that p.R420H and p.R423H reduce Kv3.3 current amplitude and cell surface expression by a dominant negative mechanism, whereas p.F448L alters the voltage-dependence of activation [2,6,16,17]. The pathological consequences of these channel alterations were validated in transgenic zebra fish and cellular models, showing deficits in locomotor control caused by suppression of the fast-spiking motor neurons excitability, defective axon pathfinding, and decreased current amplitude in the zebrafish, and impaired dendrite development and cell death in the mouse [14,18–20].

SCA13 disease prevalence among the dominant ataxias has been estimated approximately 1.5% in Europe, which is comparable to the prevalence of other non-polyQ SCA types such as SCA11, 19, 23, and 28 [21–25]. In American pedigrees of mixed ethnicity, the prevalence is estimated to be less than 1% [26]. SCA13 has not yet been reported in China [27,28].

In this study, we aimed to identify novel mutations, to get more insight in genotype-phenotype correlations and to come up with an updated assessment of SCA13 disease prevalence in the Netherlands.

## Materials and Methods

### Subjects and *KCNC3* mutation screening

Two Dutch cohorts were screened: one with 316 cerebellar ataxia patients from the Department of Genetics, University of Groningen (UMCG) and the other with 532 cerebellar ataxia patients from the Department of Medical Genetics, University Medical Center Utrecht (UMCU), the complete coding region and the exon-intron boundaries of *KCNC3* (HGNC: 6235) were examined through Sanger sequencing using the ABI3700 system (Applied Biosystems). Both diagnostic cohorts contained a mixture of unknown familial and sporadic cases. Only DNA samples were included from patients in whom testing for SCA1–3, 6, 7, 12–14, and 17 had been requested. This study did not require ethical approval since all extended DNA analyses were performed by accredited diagnostic DNA laboratories. The additional tests were thus performed in line with the original diagnostic request and no ethical committee approval was necessary. Moreover, all these patients had given permission for their DNA to be used in (anonymous) studies to help develop or improve diagnostics. However, upon the identification of potential disease causing variants, the research code was opened by the staff members of the diagnostic laboratories to reveal the identity of the corresponding case. Additionally, the consulting genetic clinician or treating neurologist was requested to recruit available family members and they also communicated the final outcomes of the test with the patient and its relatives.

The primers used for *KCNC3* sequencing are listed in S1 Table. The DNA sequences were analyzed using Mutation Surveyor software (Softgenetics). All the genetic variants identified were analyzed *in silico* with Alamut software (Interactive Biosoftware) to obtain clues about pathogenicity.

### Molecular biology

Human *KCNC3* cDNA (AF055989.1) in pHELP vector was kindly provided by Gianrico Farrugia (Mayo Clinic, Rochester, Minnesota, USA). The complete *KCNC3* cDNA was amplified using primers (S2 Table) flanked by EcoRI and KpnI restriction sides on 3’and 5’ends, respectively, to facilitate subcloning into pEGFP-C1 (Clontech). Mutations were introduced by site-directed mutagenesis PCR using specific primer pairs (S2 Table). The constructs were checked for correctness by direct sequencing.

### Cell culture and transfection

HeLa cells were grown in Dulbecco’s Modified Eagle’s Medium (Invitrogen) supplemented with 10% fetal bovine serum (Invitrogen) and 1% penicillin-streptomycin (Gibco). Chinese hamster ovary (CHO-K1) were maintained in Dulbecco’s Modified Eagle Medium: Nutrient Mixture F-12 medium (Gibco) supplemented by 10% fetal bovine serum and 1% penicillin-streptomycin. All cultures were kept at 37°C incubator with 5% CO2. For immunocytochemistry and Western blot, HeLa cells were seeded onto glass coverslips and transfected with 0.5 μg pEGFP-KCNC3 WT or mutants, or onto 6 wells/plate and transfected with 2 μg of pEGFP-*KCNC3* WT or mutants, respectively, with polyethylenimine (Polysciences). For electrophysiology, CHO-K1 cells were seeded onto glass coverslips and transfected with 0.5 μg of pEGFP-*KCNC3* WT or mutants with Genius (Westburg), following the manufacturer’s instructions. No differences in channel activity were observed between Kv3.3 and EGFP-tagged Kv3.3.

### Immunocytochemistry and Western blot

Immunofluorescense and Western blot were performed as previously described [29]. Briefly, transfected HeLa cells were fixed with 4% Paraformaldehide for 15 min, blocked and permeabilized with 0.1% Trition X-100, 5% normal goat serum in PBS (phosphate buffered saline) for 30 min at room temperature. The antibodies used in immuncytochemistry were anti-Calnexin (1:200; Santa Cruz), anti-Golgin 97 (1:200; Santa Cruz) and a secondary Cy3-conjugated goat anti-rabbit (1:500; Jackson ImmunoResearch). Coverslips were mounted in Vectashield mounting medium with Dapi (Vector Labs). Images were acquired using a Leica DMI 6000 Inverted microscope (Leica Microsystems GmbH, Germany) and processed using ImageJ software (National Institutes of Health, Bethesda, MA, USA). For Western blot, transfected HeLa cells were lysated with 2% SDS (sodium dodecyl sulfate) in PBS with protease inhibitors (Roche) and analyzed by 6% SDS-PAGE. Anti-Kv3.3 (1:500; Alomone) and anti-actin (1:5000; MP Biochemicals) antibodies were used.

### Electrophysiology

Patch clamp experiments were performed as previously described (Duarri et al., 2012). In short, potassium currents were recorded in the whole-cell configuration in CHO-K1 cells transfected either with wild type or mutant pEGFP-*KCNC3* constructs. Current amplitudes were measured by pulsing from a holding potential of-90 mV to voltages ranging from-70 to +70 mV in 10 mV depolarizing steps (800 ms). To characterize the steady-state properties of activation, conductance values were calculated from peak current amplitudes and normalized to the maximum value obtained in the experiment. Normalized conductance values were plotted *versus* test voltage and data were fitted with a single Boltzmann function to provide values for the midpoint voltage (V1/2) and slope factor. Clampfit 8.2 (Axon Instruments) and SigmaPlot 11.0 (Systat Software Inc.) were used and statistical significance was determined using a two-sample Student’s t test. Average values are expressed as mean ± SEM.

## Results

### Identification of novel *KCNC3* variants

The entire coding region of *KCNC3* was successfully sequenced in 848 cases and we identified 19 genomic variations, including 2 non-coding, 11 missense, and 6 synonymous variants (Table 1). Two of the missense variants, p.R420H and p.R423H, have previously been reported to be pathogenic as these mutations cause aberrant Kv3.3 channel functioning [2,6,16,18]. The p.R420H mutation was found in four affected members from three different families. The age of onset varied from 25–50 years of age. In the first p.R420H family, two affected cousins suffered from a slowly progressive cerebellar syndrome. In the second p.R420H family, a woman manifested a rather pure cerebellar syndrome but with normal cognitive function. Her brother also showed pyramidal signs with high tone in the legs and Babinski reflexes in addition to his cerebellar ataxia. The fourth p.R420H patient appeared to be sporadic. To investigate the possibility that the three p.R420H families might be related, haplotyping was performed using markers surrounding *KCNC3*, but no common haplotype was detected (data not shown). The p.R423H mutation was only seen once and turned out to be a de novo mutation. This female patient suffered from congenital ataxia, with a spastic ataxic gait, and mild intellectual disability. The age of onset was ~2 years and she exhibited slow motor development that stabilized during adulthood.

**Table 1:** List of all identified genetic variations in *KCNC3*.

			Frequency		Amino acid conservation		Alamut prediction				
SNP number	cDNA change	Amino acid change	Number of Dutch cases	Number of EVS- EA alleles	Mammals	Kv isoforms	GVGD	SIFT	Mutation-taster	Polyphen2	Splicing
-	c.24G>T	p.S8 =	1	0	weak						no effect
-	c.123G>T	p.Q41H	5	0	weak		benign	damaging	benign	benign	no effect
-	c.188A>G	p.D63G	1	0	weak		benign	benign	benign	benign	no effect
-	c.385G>A	**p.D129N**	1	0	high	moderate	benign	damaging	damaging	benign	no effect
rs138237939	c.579C>G	p.R193 =	2	C = 3/G = 8581	moderate						no effect
rs104894699	c.1259G>A	**p.R420H**	4	0	high	high	damaging	damaging	damaging	damaging	no effect
-	c.1268G>A	**p.R423H**	1	0	high	high	damaging	damaging	damaging	damaging	no effect
-	c.1293C>T	p.F431 =	1	0	high						no effect
rs148033381	c.1429G>A	**p.D477N**	1	T = 2/C = 8598	high	weak	benign	benign	damaging	benign	no effect
-	c.1603G>A	**p.V535M**	2	0	high	high	damaging	damaging	damaging	damaging	no effect
rs2301357	c.1641G>A	p.S547 =	3	T = 34/C = 8566	high						no effect
-	c.1771A>G	**p.S591G**	5	0	moderate	moderate	benign	benign	benign	benign	no effect
-	c.1884G>A	p.A628 =	1	0	weak						no effect
-	c.1927G>A	**p.G643S**	1	0	moderate	weak	benign	benign	damaging	damaging	no effect
-	c.1934C>G	**p.P645R**	1	0	moderate	weak	benign	damaging	benign	damaging	no effect
rs189018316	c.2170+14C>T	NA	3	A = 84/G = 8514	NA						no effect
-	c.2170+14C>G	NA	1	0	NA						no effect
-	c.2236G>A	**p.D746N**	1	0	weak	weak	benign	benign	benign	damaging	no effect
-	c.2256G>A	p.A752 =	1	0	weak						no effect

EVS, Exome Variant Server; EA, European African; GVGD, Grantham variation Grantham Deviation; SIFT, Sorting intolerant from tolerant; NA, not applicable

From the other 17 genomic coding variants found, 12 have not yet been reported (see Table 1 and partly in S3 Table), including 8 missense and 4 synonymous variants. These variants were not present in the genetic database Exome Variant Server (EVS, 8500 European exomes) or Genome of the Netherlands (GoNL,500 Dutch exomes). Two missense variants, p.Q41H and p.D63G, were excluded as potentially disease-causing, as p.Q41H was also observed in unaffected relatives of the index cases and p.D63G was detected in the homozygous state and glycine was found at position 63 in the phyla. In contrast, p.D477N was only detected twice (2/8500) in the EVS database, and therefore remained of interest.

To follow-up on our findings, we assessed the pathogenicity of the remaining missense variants using the *in silico* prediction program Alamut, and compared the outcomes to the prediction outcome of the known mutations. Not surprisingly, the known missense mutations p.R420H and p.R423H were scored as ‘damaging’ by all the prediction programs (SIFT, PolyPhen2, Mutationtaster, and Align GVGD) used by Alamut (see Table 1), while another known mutation, p.F448L, was predicted to be ‘benign’ *in silico* (data not shown) but had previously proven functionally to be pathogenic [6]. For the novel missense variants, only p.V535M was predicted to be damaging by all programs, and this mutation co-segregated with the disease in two affected family members, both suffering from early-onset (age 2–3 years), slowly progressive, cerebellar ataxia with mild intellectual disability.

The variants p.D129N, p.G643S, and p.P645R were predicted to be damaging by two programs (Table 1). The variants p.D477N and p.D746N were predicted to be damaging by one program, while p.S591G was predicted to be benign by all programs (Table 1). Consequently, all the new variants were defined as unclassified variants. These variants were detected in apparently sporadic cases, with the exception of p.S591G, which was familial in 3 out of the 5 identified cases. In fact, two cases turned out to be directly related (second family). In the first family, two sibs suffered from a slowly progressive cerebellar ataxia with spastic ataxic gait that presented at a young age (~10–15 years). Their father, who also carried the p.S591G variation, did not demonstrate any clinical symptoms at age 65, which may be indicative for a potentially very late disease onset or for non-penetrance. Additionally, this could also imply that this variant is not the disease causing variant in this family. However, in the second family, the p.S591G variation was detected in two affected brothers (treated as unrelated referrals in each of the genetic centers), with a late age-of-onset (55 and >70 years). One brother suffered from a slowly progressive cerebellar syndrome with no additional reported features, whereas the other exhibited a slowly progressive spastic ataxic gait and polyneuropathy. Unfortunately, no additional family members were available for testing. For the remaining two isolated p.S591G patients, one showed a very mild spastic ataxia syndrome that presented around age 50 years, and the other suffered from slowly progressive cerebellar ataxia with intention tremor and polyneuropathy at age 50 years. The p.D129N case was diagnosed at age 20 years, with severe cerebellar ataxia, dysarthria and intellectual disability, with a rapid progression after 25 years of age. Detailed clinical information was not available for the other cases.

The synonymous variants p.S8 = , p.F431 = and p.A752 = , and the non-coding c.2170+14C>G variant did not affect potential splice sites, whereas p.A628 = was predicted to potentially activate a cryptic splice site. However, an *in vitro* splicing assay did not show any defect (data not shown).

### Functional assessment of the novel *KCNC3* variations on Kv3.3 channel trafficking and function

Alamut predicted p.V535M to be the only pathogenic mutation and gave inconclusive results or benign outcome on the other missense variants, but because of the reporting of functional proof of p.F448L being pathogenic whilst the prediction was benign, we decided to further analyze all these novel *KCNC3* variants for Kv3.3’s protein subcellular localization (including p.R420H and p.R423H) and function.

**Fig 1:**
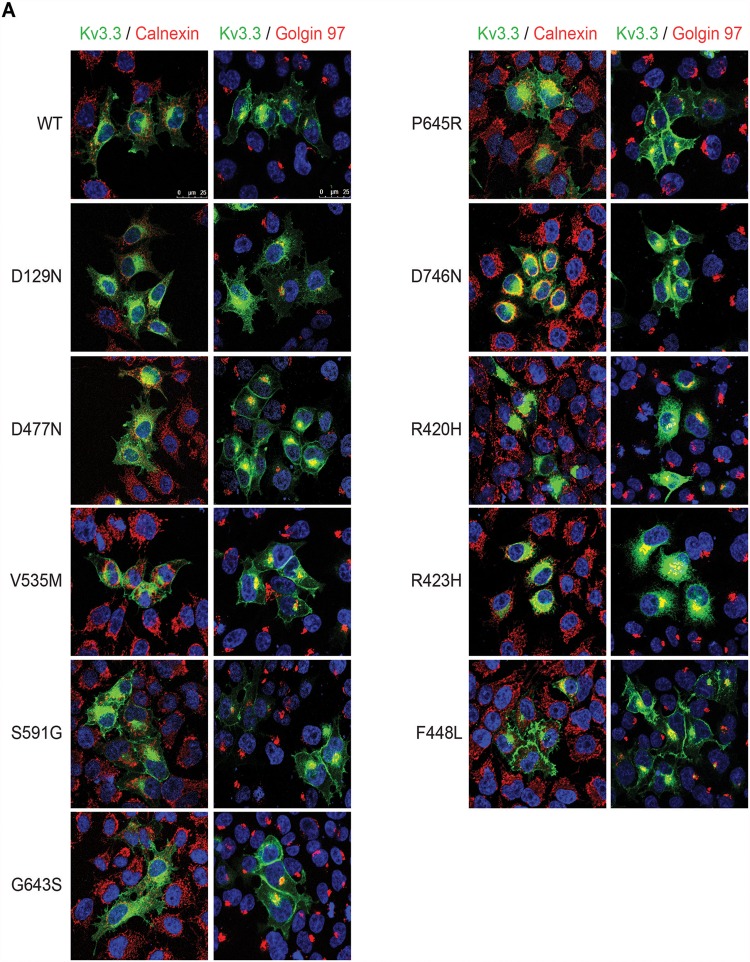
Subcellular localization and trafficking of Kv3.3 WT and mutants. Confocal images showing HeLa cells expressing EGFP-Kv4.3 WT or carrying the genetic variants (green) stained with anti-Calnexin (endoplasmic reticulum) and anti-Golgin97 (Golgi apparatus) (red), and Dapi (nucleus; blue). Scale bar, 25 μm.

**Fig 2:**
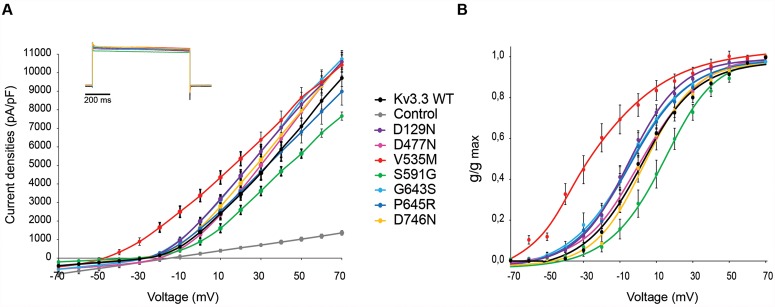
D129N, V535M and S591G variants affect the functional channel properties of Kv3.3. A) Potassium currents recorded from CHO-K1 cells expressing Kv3.3 WT or mutants. Graph shows current amplitudes evoked by pulsing from-70 mV to +70 mV in 10 mV increments and plotted *versus* voltage for WT (black), D129N (violet), D477N (pink), V535M (red), S591G (green), G643S (light blue), P645R (dark blue), D746N (yellow) and control untransfected cells (grey). Data are provided as mean ± SEM, *n* = 10. Inset: Representative current traces obtained at +70 mV scaled and overlaid for Kv3.3 WT and mutants. B) Normalized conductance values were plotted *versus* voltage and are shown as mean ± SEM. Data were fitted with a Boltzmann function to obtain the V_1/2_ of activation and the slope factor (Table 2).

First, we characterized the trafficking properties of the various Kv3.3 channels in transiently transfected HeLa cells by immunocytochemistry (Fig. 1A). Wild type (WT) Kv3.3 and channels carrying the p.D129N, p.D477N, p.V535M, p.S591G, p.G643S, p.P645R and p.D746N variants were located partially at the plasma membrane and exhibited different degrees of co-localization with the specific markers for endoplasmatic reticulum (ER) and Golgi apparatus (GA), Calnexin and Golgin-97, respectively (Fig. 2A). In contrast, the p.R420H and p.R423H mutant Kv3.3 channels showed ER retention, as described previously [17,30].

**Table 2:** Overview of Kv3.3 channel properties.

	Current density +70 mV(pA) *n* = 10	V_1/2_ Activation (mV)	Slope (mV)
**WT Kv3.3**	9711 ± 453	1.46 ± 2.2 (10)	15.5 ± 1
**p.D129N**	10588 ± 528	-7.79 ± 2.4* (14)	13.4 ± 1.3
**p.D477N**	10583 ± 371	0.79 ± 3 (10)	13,5 ± 0.9
**p.V535M**	10417 ± 589	-29.6 ± 1.0**** (18)	18.2 ± 0.8*
**p.S591G**	7661 ± 223**	17.8 ± 3.1* (16)	12.8 ± 1.2*
**p.G643S**	10736 ± 468	-4.9 ± 2.8 (10)	15.6 ± 1.5
**p.P645R**	8986 ± 733	-4.26 ± 2.3 (10)	15.9 ± 1.1
**p.D746N**	10551 ± 538	3.3 ± 1.0 (10)	13.7 ± 0.4

Statistical analysis *t*-test:

* p<0.05;

** p<0.001;

*** p<0.0001;

**** p<0.00001.

Values are provided as mean ± SEM (*n*).

Secondly, we investigated whether the novel variants would affect the functional properties of the Kv3.3 channel. Whole-cell patch-clamp experiments were performed in CHO-K1 cells expressing either WT or Kv3.3 carrying the various novel variants (Fig. 2). We confirmed significant loss of Kv3.3 channel activity due to the p.R420H and p.R423H mutations and a shift in activation curve towards the hyperpolarized direction for p.F448L mutant Kv3.3 (data not shown), as previously described [2,6,16]. We observed that only the Kv3.3 channel carrying the p.S591G variant exhibited a significantly reduced channel activity compared with WT Kv3.3 (22.2% loss at +70 mV) (Fig. 1A, Table 2); this was not observed for any of the other variants.

To characterize the steady-state of activation, normalized conductance values were plotted *versus* voltage (Fig. 2B). Here, the p.D129N and p.V535M variants shifted the voltage dependence of activation (V1/2 act) to more hyperpolarized voltages by-6 mV and-28 mV, respectively, with no changes in the slope factor (Fig. 2B, Table 2). In contrast, p.S591G variant shifted the V1/2 act to more depolarized voltages by +16 mV, with a slightly reduction of the slope factor. The remaining variants did not significantly alter the channel activation properties of Kv3.3 (Table 2).

We observed a slow and variable rate of Kv3.3 channel inactivation in CHO-K1 cells (see insert in Fig. 2A). We were therefore unable to assess any significant difference in inactivation between the WT and the mutant channels.

## Discussion

In this study, we screened a total of 848 Dutch cerebellar ataxia patients for mutations in *KCNC3*, in order to identify novel SCA13 pathogenic mutations, to gain insights into their mode of pathogenesis, and to establish a crude estimate of SCA13 disease prevalence in the Dutch ataxia population. We combined *in silico* prediction, co-segregation analysis (when possible), and functional analysis. Using this strategy, we identified at least one novel missense mutation in *KCNC3* that causes SCA13: p.V535M, as well as the previously reported p.R420H and p.R423H. Additionally, p.D129N and p.S591G might be novel SCA13 mutations as well but could still be rare benign variants. These three or five mutations were identified in five or eleven independently referred index cases, yielding a disease prevalence of SCA13 of ~ 0.6–1.3% (5 or 11/848) in the Netherlands. This makes SCA13 one of the rarer SCA types in the Netherlands. If we imply that the p.S591G is a mutation, and these five p.S591G cases are in fact (distantly) related a founder effect would even lower the SCA13 disease prevalence.

Due to the limitations of *in silico* prediction programs and the lack of additional family members to test for co-segregation, functional studies are needed to establish the pathogenicity of identified variants. All discussed variants change amino acids that are 100% conserved across the phyla (p.D129, located in the N-terminal cytoplasmatic tail; and p.V535, located in the S6 domain), or that are conserved throughout mammals (p.S591, in the initial part of the C-terminal cytoplasmatic tail), or that are fully conserved among other members of the human Kv3 family (p.V535 and p.S591) (S1 Fig.). These amino acids apparently play a crucial role in Kv3.3 channel functioning, as shown in our functional assays. Additionally, p.D129 is conserved in family member Kv3.1, which is predicted to assemble with Kv3.3 to form heterotetramer channels [17]. Similarly, our functional work showed that those variants, which were moderately or weakly conserved and predicted to be benign by more than two programs in Alamut, exhibited normal Kv3.3 channel function.

Previous studies suggested a correlation between the clinical phenotype and the respective genotypes, through the differential effects of the mutations on Kv3.3 channel function, as was determined by electrophysiological experiments in oocytes [2,6,16,17]. Altered channel gating (hyperpolarized shift in activation) was observed for both p.R423H and p.F448L mutant Kv3.3 and correlated strongly with an early disease onset, whereas suppression of Kv3.3 channel activity caused by p.R420H corresponded with a late age-of-onset. How the trafficking deficits of both p.R420H and p.R423H mutant Kv3.3 channels observed in cell models [17,30] contribute to the disease phenotype remains to be elucidated. Our results support this phenotype-genotype correlation, as p.V535M mutant Kv3.3 channels also exhibited a shift in activation towards more hyperpolarized voltages that correlated with infant/young-onset of the disease. Additionally, all patients carrying the p.R423H and p.V535M mutations exhibited mild intellectual disability as a common feature. Notably, the p.D129N variant was also associated with a hyperpolarized shift in channel activation and young age of onset. In contrast, reduced Kv3.3 channel activity and a depolarized shift in activation caused by p.S591G was associated with a spastic ataxic gait in all cases. This suggests that the presence and/or the direction of the shift in the voltage-dependent activation may be a crucial component in determining the age of onset and nature of the clinical symptoms of SCA13. However, this correlation is not always easy to establish and may not hold for other ataxias caused by mutations in voltage-gated potassium channels, including SCA19 and episodic ataxia type 1 [29,31].

Given previous work that showed that p.R420H, p.R423H, and p.F448L mutant subunits exhibited a dominant effect on wild type Kv3.3 channel activity and gating [2,6,16,20], we hypothesize that the p.V535M mutant subunits may also dominantly affect wild type Kv3.3 function, but this prediction warrants further functional studies. Additionally, we need to collect more family members of the cases carrying the p.D129N and p.S391G variants to fully confirm their pathogeneity.

Although we demonstrated that many Kv3.3 variants do not alter the channel function or the cellular localization in HeLa or CHO-K1 cells, most of them are located in the C-terminus of the protein. Since the C-terminal domain seems to target Kv3.3 channels to the distal dendrites of pyramidal neurons [32], we cannot exclude the possibility that one or more other C-terminally located variants may alter the subcellular localization in neurons and consequently cause neuronal dysfunction and SCA13.

In conclusion, SCA13 is a rare SCA genotype in the Netherlands. Our strategy in two large patient cohorts have identified at least one novel SCA13 mutation, p.V535M, and two potential additional ones; p.D129N and p.S391G. We have also provided more insight into the existing genotype-phenotype correlations through the effects of the mutation and variations on channel gating and function. Furthermore, we propose that intellectual disability and spastic ataxic syndrome are part of the clinical spectrum of SCA13. The upcoming introduction of targeted resequencing of all SCA genes (rare and more common types) in routine diagnostics will undoubtedly lead to the identification of many more variants of unknown clinical significance. For these variants, functional studies will be of the utmost importance.

## Supporting Information

S1 Fig SCA13 mutation location and amino acid conservation. (DOC)

S1 Table Primer list used for *KCNC3* sequencing. (DOC)

S2 Table Primers pairs used to generate *KCNC3* mutants and to subclone *KCNC3* cDNA into pEGFP-N1. (DOC)

S3 Table Genetic and clinical information of newly identified *KCND3* missense variants. (DOC)
