# Supplementary material for: Functional Analysis Helps to Define KCNC3 Mutational Spectrum in Dutch Ataxia Cases
Source: PLoS One. 2015 Mar 10;10(3):e0116599. doi: 10.1371/journal.pone.0116599 (PMC4355074; doi:10.1371/journal.pone.0116599)
Supplement: S1 Fig — (DOC) [file pone.0116599.s001.doc]

**Figure S1. SCA13 mutation location and amino acid conservation**.

**
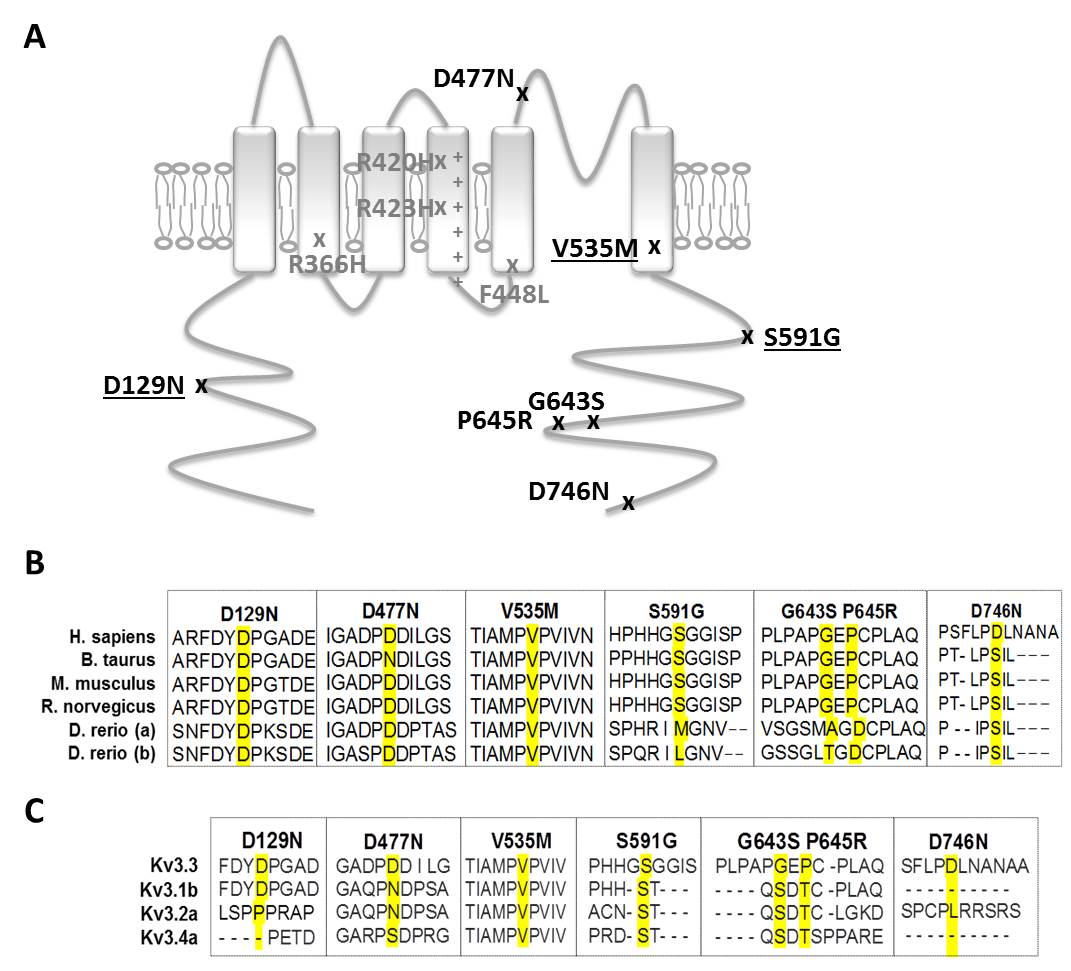
**

**Figure S1.** A) Localization of the Kv3.3 variants. The 7 new variants are designated in black; the pathogenic mutations linked to SCA13 are black underlined; and the SCA13 mutants previously reported are in grey [1,2]. B) Comparison of Kv3.3 protein sequence (the positions of the variants are highlighted in yellow) between *H. sapiens* KCNC3 (NP_004968), *B. taurus* KCNC3 (XP_003587351.2), *M. musculus* kcnc3 (NP_032448.2), *R. norvegicus* kcnc3 (NP_446449.2), *D. rerio* kcnc3a (NP_001182169.1) and kcnc3b (NP_001182170.1)). C) Protein sequence alignment of human Kv3.3 (NP_004968), Kv3.1b (NP_004967), Kv3.2a (NP_631874) and Kv3.4a (NP_004969).

References

1. Waters MF, Minassian NA, Stevanin G, Figueroa KP, Bannister JP, et al. (2006) Mutations in voltage-gated potassium channel KCNC3 cause degenerative and developmental central nervous system phenotypes. Nat Genet 38: 447-451.

2. Figueroa KP, Minassian NA, Stevanin G, Waters M, Garibyan V, et al. (2010) KCNC3: Phenotype, mutations, channel biophysics-a study of 260 familial ataxia patients. Hum Mutat 31: 191-196.
