# Supplementary material for: Functional Analysis Helps to Define KCNC3 Mutational Spectrum in Dutch Ataxia Cases
Source: PLoS One. 2015 Mar 10;10(3):e0116599. doi: 10.1371/journal.pone.0116599 (PMC4355074; doi:10.1371/journal.pone.0116599)
Supplement: S1 Table — (DOC) [file pone.0116599.s002.doc]

**Table S1. Primer list used for *KCNC3* sequencing**

| **Amplicon** | **Forward primer** | **Reverse primer** |
| --- | --- | --- |
| 1-1 | GTGTCCCCTCCTCCCTCTAC | AAGGACGAGACGCAGACTGA |
| 1-2 | TTGGTTCCTCTCCCCTAAGC | TCGAGCGGTACGTCTCATGG |
| 1-3 | TCAGTCTGCGTCTCGTCCTT | TCAAAGAAGAACTCGTCGGC |
| 1-4 | CCATGAGACGTACCGCTCGA | GGAAGCAGAGGCGCTTGAG |
| 1-5 | CTGCTGCTGGATGACCTACC | TGAGAAGCCTAGAGGGACCC |
| 2-1 | GATGCCTAGGTCACCTCT | GTCGATGATGTTGAGGCTG |
| 2-2 | CCCAGACAAGGTGGAGTTT | CGTAGTAAATCATGGTGGCGA |
| 2-3 | CTGCTGCTCATCATCTTCCT | TTGCAGTAGTTGGGCGAG |
| 2-4 | CAAGAAGAAGAACAAACACATCC | CAGCTACCTCCCCAGTC |
| 3 | CTCTCTCCTTTGTCTCTCTGT | GGTCCCAGGGGATCAGTA |
| 4 | TTCCTCACCACTGACCCTTC | GGTTAGTCAGGCAGGAGTGG |
| 5 | CCCGTGACTCTGTGTATTTCT | AGGCTCTCACAGGCATC |
