# Supplementary material for: Functional Analysis Helps to Define KCNC3 Mutational Spectrum in Dutch Ataxia Cases
Source: PLoS One. 2015 Mar 10;10(3):e0116599. doi: 10.1371/journal.pone.0116599 (PMC4355074; doi:10.1371/journal.pone.0116599)
Supplement: S2 Table — (DOC) [file pone.0116599.s003.doc]

**Table S2.** **Primers pairs used to generate *KCNC3* mutants and to subclone *KCNC3* cDNA into pEGFP-N1.**

| **Mutation** |  | **Mutagenesis primers** |
| --- | --- | --- |
| p.D129N | FOR | CGCTTCGACTACAACCCGGGCGCCG |
|  | REV | CGGCGCCCGGGTTGTAGTCGAAGCG |
| p.R420H | FOR | GTCCGCTTCGTCCACATCCTGCGCATC |
|  | REV | GATGCGCAGGATGTGGACGAAGCGGAC |
| p.R423H | FOR | GTCCGCATCCTGCACATCTTCAAGCTG |
|  | REV | CAGCTTGAAGATGTGCAGGATGCGGAC |
| p.F448L | FOR | CAGCACCAACGAGTTACTGCTGCTCATCATC |
|  | REV | GATGATGAGCAGCAGTAACTCGTTGGTGCTG |
| p.D477N | FOR | GGCGCCGACCCCAATGACATCCTGG |
|  | REV | CCAGGATGTCATTGGGGTCGGCGCC |
| p.V535M | FOR | CATCGCCATGCCTATGCCCGTCATTG |
|  | REV | CAATGACGGGCATAGGCATGGCGATG |
| p.S591G | FOR | CCGCACCACGGCGGCGGGGGCATCAG |
|  | REV | CTGATGCCCCCGCCGCCGTGGTGCGG |
| p.G643S | FOR | CTGCCAGCCCCCAGCGAGCCTTGCC |
|  | REV | GGCAAGGCTCGCTGGGGGCTGGCAG |
| p.P645R | FOR | CCCCCGGCGAGCGTTGCCCGTTGGC |
|  | REV | GCCAACGGGCAACGCTCGCCGGGGG |
| p.D746N | FOR | CAAGCTTCTTGCCCAACCTCAACGCCAAC |
|  | REV | GTTGGCGTTGAGGTTGGGCAAGAAGCTTG |
| **Kv3.3** |  | **PCR for pEGFP-C1** |
| Kv3.3-C1-For | FOR | GCCGAATTCCATGCTGAGCTCAGTCTGCG |
| Kv3.3-C1-Rev | REV | CGG GGT ACC CTA GGG GGA TAT CCA GGC CGC |
