# Supplementary material for: Functional Analysis Helps to Define KCNC3 Mutational Spectrum in Dutch Ataxia Cases
Source: PLoS One. 2015 Mar 10;10(3):e0116599. doi: 10.1371/journal.pone.0116599 (PMC4355074; doi:10.1371/journal.pone.0116599)
Supplement: S3 Table — (DOC) [file pone.0116599.s004.doc]

**Table S3**: **Genetic and clinical information of newly identified *KCND3* missense variants**

| **Mutation** | **Conservation** | **Cellular localization** | **Channel functional deficit** | **Onset (years)** | **Familial SCA13/Sporadic** | **Clinical phenotype** |
| --- | --- | --- | --- | --- | --- | --- |
| **p.D129N** | Conserved | PM/GA | Left shift activation | 20 | Sporadic | severe cerebellar ataxia, dysarthria, intellectual disability |
| **p.R420H** | Conserved | ER | Reduced current^1^ | 25-50 | Familial | slowly progressive cerebellarsyndrome, pyramidal signs |
| **p.R423H** | Conserved | ER | Reduced current^2^ | 2 | Sporadic | congenital ataxia, spastic ataxic gait, mild intellectual disability, slow motor development |
| **p.D477N** | Conserved | PM/GA | = WT | - | - | - |
| **p.V535M** | Conserved | PM/GA | Left shift activation, increased slope | 2-3 | Familial | slowly progressive cerebellar ataxia, mild intellectual disability |
| **p.S591G** | Mammals | PM/GA | Reduced current, right shift activation, reduced slope | 10-70 | Familial/ Sporadic | slowly progressive cerebellar ataxia, spastic ataxic gait, intention tremor, polyneuropathy |
| **p.G643S** | Mammals | PM/GA | = WT | - | - | - |
| **p.P645R** | Mammals | PM/ER/GA | = WT | - | - | - |
| **p.D746N** | Not conserved | PM/ER/GA | = WT | - | - | - |

PM, plasma membrane; ER, Endoplasmic reticulum; GA, Golgi apparatus. ^1^Waters et al., 2006; ^2^Figueroa et al., 2010
